# Editorial: Understanding cyberbullying from various perspectives, volume II

**DOI:** 10.3389/fpsyg.2026.1882017

**Published:** 2026-06-30

**Authors:** Shruti Soudi

**Affiliations:** Department of Psychology, People's Education Society (PES) University, Bengaluru, India

**Keywords:** cyberbullying, family dynamic, impact on learning, neuroscience, personality

“Ā·no·bhadrāḥ·kratavo·yantu·viśvataḥ” is a very famous Vedic philosophy with which we began the second volume of “*Understanding Cyberbullying from Various Perspectives*.” The crux of the quote matches our intent and outcome of this scholarly project after a year full of contributions, reviews and reflection. In a world full of unquenching thirst for dopamine, where the chase for instant gratification often ignores moral, biological, social, and even legal boundaries, the digital age stands as both a marvel and a menace. It has revolutionized how young people connect, learn, and express themselves, offering unprecedented opportunities for creativity and community.

Yet, there is a flip side for this, cyberbullying. Unlike the cruelty of face-to-face bullying, online aggression is relentless, often masked in anonymity, and magnified by the boundless reach of digital platforms. Its sting lingers longer, its wounds cut deeper, and its prevention demands far more than traditional approaches.

What makes cyberbullying especially deceptive is its persistence as it follows victims into their homes, their private spaces, their late-night scrolling. The very tools designed to empower and entertain can become weapons of humiliation and harm. And because the digital world rarely sleeps, the cycle of abuse can feel endless.

This paradox of technology as both liberator and oppressor force us to confront urgent questions about responsibility, empathy, and the kind of digital culture we want to build.

This second volume of “*Understanding Cyberbullying from Various Perspectives*” brings together seven studies that explore the issue through psychological, social, educational, and even neurobiological lenses. By including research from diverse cultural contexts, the volume emphasizes the importance of cross-cultural sensitivity in both theory and practice.

A recurring theme across the contributions is the role of family dynamics. One study shows that parental psychological control can indirectly fuel cyberbullying by disrupting sleep and encouraging moral disengagement. Interestingly, this effect is stronger in older adolescents, pointing to developmental differences that interventions must take into account. Another study highlights how the pursuit of popularity, often reinforced by parents and peers, can increase cyber aggression, while genuine social acceptance reduces it.

The volume also throws a light on bystanders, who are often overlooked in discussions of cyberbullying. Research with Chinese college students reveals that parenting styles influence whether bystanders support bullying: rejection fosters support for aggression, while emotional warmth reduces it. This underscores the need for interventions that consider not just bullies and victims, but also the wider social environment.

From an educational perspective, findings from Spanish schools show that cyberbullying doesn't just harm emotional well-being it also undermines learning. Victims and perpetrators alike struggle with procrastination, poor planning, and superficial learning, with cyberbullying having even stronger negative effects than traditional bullying.

Personality traits also play a role. One study distinguishes between grandiose and vulnerable narcissism in predicting cyberbullying and trolling. Vulnerable narcissism consistently drives online aggression, while grandiose narcissism is more situational linked to motives like revenge or reward. This nuance highlights the importance of tailoring interventions to individual psychological profiles.

Adding to the global scope, research on Indian adolescents explores how parental rules shape experiences of cyber-victimization and mental health. The findings reveal a dual role: parental regulation can be protective, but if overly restrictive, it may backfire. This points to the need for culturally responsive approaches that respect local values and parenting practices.

Finally, the volume ventures into neurobiological research, examining how victims regulate emotions in response to online aggression. By connecting psychological theory with brain processes, this work opens new possibilities for interventions that strengthen emotional regulation at both cognitive and neurological levels.

Methodologically, the studies employ innovative approaches such as moderated mediation models and longitudinal designs that capture the dynamic, context-dependent nature of cyberbullying. Together, they highlight the importance of global collaboration and comparative research to design interventions that are universally informed yet locally adaptable.

In sum, this volume enriches the conversation around cyberbullying by weaving together insights from psychology, education, sociology, and neuroscience. It offers practical implications for parents, educators, policymakers, and mental health professionals, while calling for future research that continues to explore underrepresented populations and leverages technology to promote positive digital citizenship.

While this volume deepens our understanding of cyberbullying through diverse disciplinary and cultural lenses, the phenomenon continues to evolve alongside technology. Future research must anticipate emerging challenges and opportunities.

One promising direction is the role of artificial intelligence and machine learning in detecting and preventing cyberbullying. As platforms increasingly rely on automated moderation, scholars must examine not only how effectively these systems identify harmful behavior but also how they can be refined to avoid bias and misinterpretation. The integration of AI into prevention strategies could mark a new era of proactive digital safety.

Equally important is the recognition that risks differ across digital spaces. Social media, gaming platforms, and messaging apps each foster unique dynamics that shape how bullying unfolds. Understanding these platform-specific vulnerabilities will be crucial for designing interventions that are both targeted and effective.

Another area of growing interest is the positive influence of bystanders. Research can explore how digital tools and educational initiatives might empower witnesses to act with empathy, resilience, and prosocial intent. Encouraging bystander intervention not only disrupts harmful behavior but also helps cultivate a culture of thriving digital citizenship where online communities are built on dignity, support, and shared responsibility.

